# From A to Z: a potential role for grid cells in spatial navigation

**DOI:** 10.1186/2042-1001-2-6

**Published:** 2012-05-30

**Authors:** Caswell Barry, Daniel Bush

**Affiliations:** 1 UCL Inst of Cognitive Neuroscience, London, UK; 2 UCL Inst of Neurology, London, UK; 3 UCL Inst of Behavioural Neuroscience, London, UK

## Abstract

Since their discovery, the strikingly regular and spatially stable firing of entorhinal grid cells has attracted the attention of experimentalists and theoreticians alike. The bulk of this work has focused either on the assumption that the principal role of grid cells is to support path integration or the extent to which their multiple firing locations can drive the sparse activity of hippocampal place cells. Here, we propose that grid cells are best understood as part of a network that combines self-motion and environmental cues to accurately track an animal’s location in space. Furthermore, that grid cells - more so than place cells - efficiently encode self-location in allocentric coordinates. Finally, that the regular structure of grid firing fields represents information about the relative structure of space and, as such, may be used to guide goal directed navigation.

## Introduction

**Figure 1:**
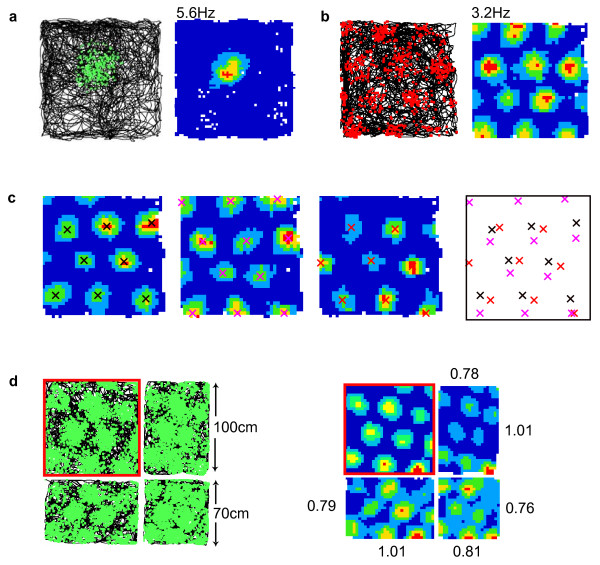
**Single unit recordings made from the hippocampal formation. a)** CA1 place cell recorded from a rat. The left-hand figure shows the raw data: the black line being the animal’s path as it foraged for rice in a 1 m^2^ arena for 20 minutes; superimposed green dots indicating the animal’s location each time the place cell fired an action potential. Right, the same data processed to show firing rate (number of spike divided by dwell time) per spatial bin. Red indicates bins with high firing rate and blue indicates low firing rate, white bins are unvisited, and peak firing rate is shown above the map. **b)** Raw data and corresponding rate map for a single mEC grid cell showing the multiple firing fields arranged in a hexagonal lattice. **c)** Three co-recorded grid cells, the center of each firing field indicated by a cross with different colors corresponding to each cell. The firing pattern of each cell is effectively a translation of the other co-recorded cells as shown by superposition of the crosses (right). **d)** Changes made to the geometry of a familiar environment cause grid cell firing to be distorted (rescale) demonstrating that grid firing is, at least, partially controlled by environmental cues, in this case the location of the arena’s walls. Raw data are shown on the left and the corresponding rate maps on the right. The rat was familiar with the 1 m^2^ arena (outlined in red). Changing the shape of the familiar arena by sliding the walls past each other produced a commensurate change in the scale of grid firing. For example, shortening the x-axis to 70 cm from 100 cm (top right) caused grid firing in the x-axis to reduce to 78% of its previous scale, while grid scale in the Y-axis was relatively unaffected. Numbers next to the rate maps indicate the proportional change in grid scale measured along that axis (figure adapted from reference [28]).

Half a century’s worth of research has established, beyond doubt, the role of the hippocampal formation in memory. Damage to the human hippocampus and surrounding cortex results in profound amnesia for events occurring after the insult and also for those occurring beforehand in a temporally graded fashion [1,2]. Such patients exhibit impaired spatial cognition, have difficulty navigating [3], remembering the relative location of objects [4], and even visualizing imagined scenes [5]. In model organisms, such as the rat, lesions of the hippocampal formation produce similar deficits, including an impaired ability to navigate [6] and failure to recognize novel spatial arrangements [7]. Single unit recordings made in the early 1970s first intimated a neural basis for these functions in the form of place cells (Figure 1a), hippocampal pyramidal neurons with spatially localized firing fields (place fields) [8]. Prompted by this discovery, O’Keefe and Nadel proposed that place cells constitute part of a hippocampal network which functions as a cognitive map; representing an animal’s location within its environment relative to other objects and, hence, enabling flexible navigation strategies, including novel short-cuts and detours [9]. Furthermore, they suggested that this cognitive map is the basis of human episodic memory, the spatial framework being embellished to encode the content of specific events.

While place cells provide an undeniably spatial signal, attempts to derive models that would support flexible navigation proved difficult for several reasons. First, it was initially believed that place cells simply encoded an animal’s current location, with no capacity to represent the route to, or site of, a navigational goal. Second, the sparsely distributed, irregular place fields of an ensemble of place cells do not obviously convey information about the relative proximity of those fields in a given environment. Direct connectivity between place cells, such as that found in CA3, could encode the distance between place fields [10]. However, the synaptic weight matrix would have to be learned for each new environment and could not support accurate navigation across unvisited areas, a feat that many animals, including rodents, are capable of [11]. Both these barriers now seem to be falling away. The increasingly well understood phenomena of preplay and replay in ensembles of hippocampal place cells [12,13] together with the discovery of entorhinal grid cells with periodic spatial firing fields [14,15], indicate that the representation of space in the hippocampal formation is both non-local and spatially structured. Here we argue that grid cells, and not place cells, principally encode self-location in allocentric coordinates and, furthermore, that they also represent the relative proximity of spaces in an animal’s environment. Finally, because grid cells encode spatial information in this way, they are likely to be a key part of a network supporting vector based navigation, which would, for example, enable an animal to travel the shortest route between its current location and a distant goal.

## Review

### Properties of grid cells

Grid cells exhibit a strikingly regular, spatially stable, firing pattern of circular fields arranged in a hexagonal lattice that covers the environment [14,15] (Figure 1b). Since their initial discovery in the entorhinal cortex of rats, they have also been identified in mice, humans and bats [14-18]. Like the hippocampus, the entorhinal cortex can be subdivided on the basis of morphology and connectivity, the principal distinction being between the medial and lateral entorhinal cortices (mEC and lEC) [19]. The lEC receives primarily unimodal sensory information from perirhinal cortex as well as frontal, piriform and olfactory cortices, while the mEC receives spatial information from the multimodal association areas, specifically retrosplenial, parietal and occipital cortices [19]. These two pathways are often characterized as the ‘what’ and ‘where’ processing streams respectively [20]. Grid cells are not found in the lEC, being limited to the mEC [15,21], where they are most numerous. Although grid cells were initially identified in layer II of mEC, subsequent work has found them in layer III and the deep layers V and VI [22], as well as the para- and post- subiculum [23]. Grid cells from these other areas, unlike the layer II cells, often exhibit firing modulated by the animal’s head direction and are co-localized with head direction cells that solely encode direction of facing [22] and border cells that encode proximity to environmental barriers [24]. Importantly, while mEC layers II and III project to the hippocampus, the deeper layers V and VI receive return projections from CA1 and subiculum [19] and, subsequently, project back to the shallow layers, placing grid cells within a processing loop that encompasses most of the hippocampal formation [19].

**Figure 2:**
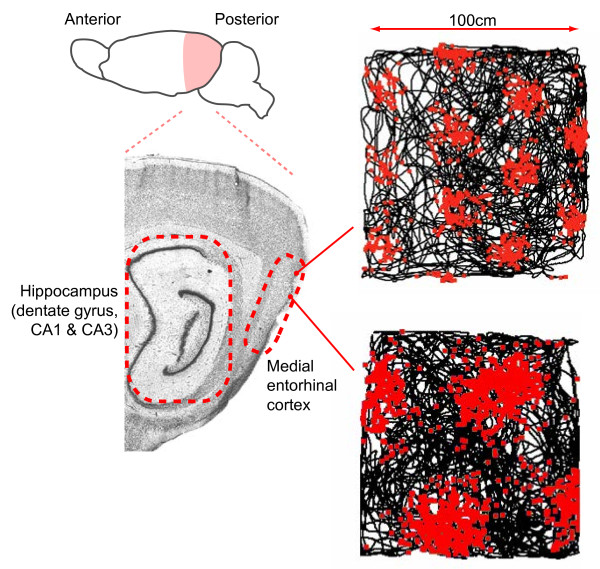
**Grid scale increases along a dorso-ventral gradient in the mEC.** Two grid cells recorded from the same animal but at different times are shown, both cells were recorded in a familiar 1 m^2^ arena. Approximate recording locations in the mEC are indicated. The more ventral cell exhibits a considerably larger size of firing fields and distance between firing fields than the dorsal cell.

The scale of the grid pattern, measured as the distance between neighboring peaks, increases along the dorso-ventral mEC gradient, mirroring a similar trend in hippocampal place fields [15,25]. The smallest, most dorsal, scale is typically 20 to 25 cm in the rat, reaching in excess of several meters in the intermediate region of the gradient [15,26] (Figure 2). This may explain how this remarkable pattern was missed by early electrophysiology studies, which targeted ventral mEC and found only broadly tuned spatial firing (for example, [27]). Interestingly, grid scale increases in discontinuous increments and the increment ratio, at least between the smaller scales, is constant [28]. Grid cells recorded from the same electrode, which are, therefore, proximate in the brain, typically have a common scale and orientation but a random offset relative to each other and the environment [15]. As such, their firing patterns are effectively identical translations of one another and a small number of cells will ‘tile’ the complete environment (Figure 1c). It also appears that grids of different scale recorded ipsilaterally have a common orientation, such that the hexagonal arrangement of their firing fields share the same three axes, albeit with some localized distortions [15,28,29].

### Mechanisms of grid field formation

Due to their invariant spatial metric, theoretical models of grid cell activity have almost exclusively described their firing in terms of a system that integrates idiothetic cues in order to update self-location. In fact, it is hard to see how such a regular pattern that is coherent between neighboring cells could be otherwise produced. However, these models differ significantly in the manner by which they account for the formation of the grid field; either by continuous attractor dynamics or oscillatory interference. Attractor models hypothesize that grid cell activity reflects a ‘packet’ or ‘packets’ of localized excitation on a flat energy landscape provided by recurrent connections, and that this activation can be smoothly shifted by translational input from speed modulated head direction or conjunctive cells [30-32]. Conversely, oscillatory interference models posit that grid firing is generated by interference between velocity controlled oscillators (VCOs) – individual cells or small networks that increase their firing frequency according to the speed of movement in a preferred direction – and a baseline oscillation [33-36]. The phase difference between these oscillations then reflects displacement in the preferred direction of the VCOs. Hence, interference between the baseline oscillation and VCOs with preferred directions that differ by multiples of 60° produces periodic spatial tuning with six fold rotational symmetry.

Evidence in favor of the attractor model comes from the existence of the requisite speed modulated head direction and conjunctive cells in mEC, post- and para- subiculum, which also appear to have the necessary anatomical connections to grid cells in those regions [22,37]. The observed topographical clustering of grid scales and orientation is also a necessary component of attractor models [28]. Criticism of this class of model is generally focused on the apparent lack of recurrent excitatory connections between principal cells in layer II mEC, although contradictory reports do exist and attractor dynamics might be maintained in the deeper layers by recurrent inhibition, be limited to the dense entorhinal cell islands, or be located in either the pre- or para- subiculum [23,31,37-40]. Moreover, the stable operation of any attractor network requires precisely tuned synaptic weights to prevent drift or disruption of the activity packet, and it is not clear how such connectivity would be developed or maintained *in vivo*.

Evidence in support of the oscillatory interference models comes from recordings of putative velocity controlled oscillator cells in the anterior thalamus, medial septum and hippocampus that exhibit cosine directional tuning of firing frequency [41]. Similarly, inactivation of the medial septum in rodents, which generates the theta frequency oscillations that dominate the hippocampus during translational movement, quickly eliminates the grid field firing pattern [42,43]. Most compelling, though, is evidence that links the theta-band frequency of membrane potential oscillations in mEC grid cells with their scale, as predicted by the model [44,45]. Manipulations that show a change in one of these properties is accompanied by the expected change in the other are also encouraging [46,47]. The principal criticism of this class of model relates to the difficulty of maintaining precisely timed oscillations in neural circuits – as any error accumulated in the VCOs will quickly disrupt the resultant grid field [39,48]. Simulations indicate that this is not necessarily the case, however. While independent realistically noisy oscillators would quickly decohere, a population of coupled oscillators would not [36,41]. Similarly, the observation of phase precession in grid cell firing is not only explained by this class of model but also indicates that the timing of neural oscillations can be maintained with a high degree of fidelity *in vivo*[49]. Recently, recordings made from crawling fruit bats appear to present a serious challenge for the oscillatory interference models: the bats exhibit no continuous theta in the entorhinal or hippocampal LFP and have grid cells with no theta-band modulation of the spike train [18]. However, the very low movement speed and firing rates make these results difficult to interpret as they render theta-band modulation of the spike train hard to detect [50]. The same group has presented place cell recordings from flying bats but it is unclear if this data exhibit theta-band modulation because of artefacts created by the animals’ 7-8Hz wing beats [51]. Interestingly, several models of grid cell firing have recently been published which incorporate both recurrent connectivity and temporal dynamics (for example, [41,52]). Interaction between these two, possibly redundant, mechanisms may account for the disparity in results so far reported from different species.

### Path integration

So what is the function of grid cells? Or, more meaningfully, what information do grid cells encode? Again, most models and theoretical studies have focused either on the assumption that their principal function is to support path integration [30,31,34,36,53,54] or the extent to which their multiple firing locations can drive the unitary firing of place cells [55-57]. It is to the first of these points that we turn.

Path integration is a basic navigational strategy observed across a wide range of species in which an animal’s current position relative to some reference point is maintained by continually integrating the direction and distance moved according to idiothetic cues [11,58]. The experimental studies described above delineate several properties of grid cells that provide indirect evidence for an involvement in path integration. First, they are co-localized with head direction and conjunctive cells that exhibit coherent spatial tuning across different environments, such that all the information required to perform path integration is present in the local circuit [22,59]. Second, the grid field is generated rapidly in a novel environment, updated in the absence of visual input, and stable to the removal of local cues [15,59]. Finally, the spatial scale of the grid field is fixed across familiar environments and striking in its regularity, thereby providing a coherent, consistent and reliable estimate of allocentric distance travelled in a context independent fashion [15,59].

That said, grid firing does not simply track accumulated idiothetic cues, it encodes an animal’s location in allocentric space and, once established, is clearly stabilized and controlled by environmental (allothetic) cues. For example, grid firing is stable between visits to an environment, over distances and durations that are unlikely to be accurately judged on the basis of idiothetic information alone [15]. More obviously, the orientation of grid firing is controlled by movement of a single polarizing cue in an otherwise symmetrical circular environment [15]. Similarly, the spacing and regularity of the grid pattern is influenced by manipulations of the shape and position of boundaries within an animal’s environment (Figure 1d). In fact, when self-motion and environmental cues are placed in contradiction, grid firing is initially more strongly shaped by the latter [28,60].

How might environmental cues become associated with grid firing? An interesting suggestion raised by O’Keefe and Burgess [61] is that information about the location of environmental cues, such as boundaries, may reach the deep layers of mEC via projections from hippocampal place cells. Projections from deep to shallow layers would then convey this information to grid cells in layers II and III. Although the hippocampus is typically seen as being efferent to the entorhinal cortex, this idea is appealing for several reasons. First, multimodal spatial information from the mEC is only fully combined with unimodal sensory information from the lEC within the hippocampus, most likely at the level of recurrent circuitry in CA3 [21]. Second, computational considerations suggest that it may be easier to associate the multiple spatial cues that identify a specific location with a single place field rather than directly with the multiple firing locations of a grid cell [61]. Third, temporary inactivation of the hippocampus quickly causes the spatial firing of grid cells to break down [49,62]. Finally, in pre-weanling rats, grid cell firing patterns are never found before stable place cell firing has developed and normally appear later, at days p20 to 21 and p16 respectively [63,64].

It is equally probable that place cells receive input from grid cells: place cell firing in CA1 can be supported by direct entorhinal input after disruption of CA3 projections [64]; place fields convey less spatial information following lesions made to dorsal mEC [66]; and place fields appear to have access to idiothetic information that may be conveyed by the grid network [67]. So, if grid cell firing is influenced by the activity of place cells in the hippocampus and vice versa, then on what basis can we attempt to distinguish separate functions for these two regions?

Our claim is that grid cells are part of a network that enables self-motion and environmental cues to be combined to provide an optimal estimate of an animal’s location. Furthermore, that grid firing efficiently encodes this information in allocentric coordinates. In contrast, although place cell firing primarily represents allocentric self-location, the nature of that code is less efficient and appears to encode for other, non-spatial, variables. Although the repeated firing fields of a single grid cell produce an ambiguous spatial code, combinations of grid fields with different scales increasingly reduce this ambiguity. This is emphasized by numerous computational models that describe how the firing of multiple grid fields can be combined to produce the sparse firing of hippocampal place cells [55-57]. Similarly, the activity of a small number of different scaled grid cells is sufficient to uniquely specify the location of a rat and can be experimentally decoded to reveal that location [14]. Numerical analyses conducted in the Fiete lab have yielded the most complete and informative exploration of the limits of the grid code. These analyses demonstrate that grid cells can function as a residue number system, suggesting that the capacity of the network is combinatorial, growing disproportionately with the addition of units with different scales [39,68]. Under certain assumptions, maximum capacity is obtained when the combined scales have no common factors, in other words when the ratio of grid scales form a prime number sequence [68]. It is interesting to note that the reported scale increment between the smallest grid scales is around 1.65, a good approximation for the ratio between the prime numbers 5 and 3 [28]. If these assumptions are met, then with 8 to 10 distinct grid scales the mEC can uniquely locate an animal to an accuracy of a few centimeters within an area of several square kilometers; a much greater capacity than could be achieved with a similar number of place cells [68]. Furthermore, since the capacity of the grid network is greater than the typical range of a wild rat [68], this suggests a possible strategy for error correction. The combinatorial grid code is sensitive to errors in any of its differently scaled units; a small error in one or more unit will lead to a large decoding error. This is helpful in two ways: first, given the capacity of the system, the error will often decode to a position that the animal has never visited; and second, this position will probably be unrealistically far from the previous location of the animal. Sreenivasan *et al*[32] have demonstrated that, due to these properties, errors in the grid code can be constrained and corrected by a recurrent network similar to that found in CA3.

So, is the sole function of the hippocampal place cell network to support grid firing by conveying sensory cues and correcting errors? This is unlikely. The activity of hippocampal place cells appear to differ from grid cells in two important ways: it is both more variable and more sparse, and these properties suggest that place cells are able to encode information in addition to self-location. The clearest demonstration of this is that environmental manipulations which produce changes in the firing rates of place cells (rate remapping) do not affect the firing of co-recorded grid cells [59]. More generally, a considerable body of research has shown that the firing rates of individual place cells is highly variable between visits to the same location [69]. Furthermore, place cells exhibit rate remapping between environments that differ subtly [70], and can accumulate rate and spatial changes such that the place code increasingly distinguishes environments or locations that had previously been identically encoded [70-72]. Currently, it is unknown if grid cells exhibit comparable phenomena, though none have so far been reported. Taken together, these results suggest that the grid code is limited to spatial information, while hippocampal place cells are able to encode additional experiential factors by modulating the spatial code.

### Non-local coding and navigation

In addition to current position, the regularly distributed firing fields of grid cells carry information about the relative proximity of places in an environment. For example, when a rat is traveling in a single dimension, movement from one region of peak grid cell firing to another indicates displacement by some integer multiple of the grid scale. In principal, the calculations that update grid firing by integrating idiothetic cues can be reversed in order to extract the translational vector between two allocentric locations. Such a process could provide the basis for a navigation system that would enable an animal to travel directly from its current location to a non-visible goal, a task which rodents and other animals can ably perform [11]. Might a grid cell network support these navigational abilities?

Preliminary theoretical results indicate that it is possible to create a network that will extract both the distance and direction of a goal from the activity of a population of grid cells [73,74]. However, as yet, there are no published models that actually direct navigation on the basis of grid firing (see note added in proof). Entorhinal lesion studies indicate that damage to the grid network does impair an animal’s ability to reach a hidden goal but, because the entorhinal cortex is reciprocally connected with the hippocampus, it can be difficult to interpret these results. That said, lesions focused on the shallow layers of dorsal mEC eliminated spatial preference in rats trained on the Morris water maze [75]. Importantly, the animals were subsequently able to relearn the task, indicating that some degree of spatial processing was preserved. Less specific entorhinal lesions also produce deficits in the water maze and particularly impact an animal’s ability to navigate directly to the escape platform. Interestingly, the rats change strategy as a result, searching for the goal close to cues placed within the maze [76]. Lesions made to the entorhinal and parietal cortex also produce path integrative deficits in a homing task [77]. Similarly, bilateral disconnection of the entorhinal-hippocampal circuit was found to impair detection of a spatial change when familiar objects were moved relative to one another, possibly indicating a deficit in the ability to judge relative position [78]. However, contradictory results do exist, for example Burwell *et al.*[79] did not detect navigational deficits after making entorhinal lesions in rats. A likely source of the reported variability is that several of these studies were conducted before the discovery of grid cells, and lesions were made without knowledge of the precise topographical arrangement of those cells within entorhinal cortex.

Finally, accumulating electrophysiological results from the last 15 years have increasingly shown that place cells can fire non-locally, effectively encoding trajectories removed from the animal’s current location [12,13,80]. These events typically occur during hippocampal sharp waves, brief periods of activity characterized by a reduction in inhibition and transient high frequency oscillations (100 to 200 Hz ‘ripples’ [9]) in the local field potential, as well as during REM sleep. It has been known for some time that hippocampal ripples reach the entorhinal cortex [81] and preliminary results indicate that grid cells also participate in these preplay events [81]. What remains unproven is whether these events are related to task demands and might, therefore, indicate the route that an animal will subsequently follow to reach a goal. Though not directly related to preplay, recordings made from mEC while rats performed a T-maze alternation task showed that cells in this region (though not explicitly grid cells) modulated their firing according to the route that the animal was following [83]. Similar results have been noted for place cells [84]. However, in this case the authors compared the mEC modulation with co-recorded place cells and found that the entorhinal effect was larger and more informative about the animal’s future actions. fMRI studies also implicate the entorhinal cortex in navigational planning. For example, a study of London taxi drivers navigating in a virtual reality rendition of central London demonstrated that entorhinal activity positively correlated with Euclidian distance to a goal [85].

## Conclusions

It seems clear that the regular firing pattern of grid cells represent an efficient strategy for encoding self-location in allocentric coordinates. Furthermore, that grid cells and place cells form part of a network that combines idiothetic and allothetic cues to accurately track an animal’s movement through space. It is also clear that the activity of a population of grid cells encodes information about the relative structure of space. What is currently unknown is whether this metric is accessible to other structures in the brain and, if so, whether it is employed during navigation. Existing results and theoretical models suggest this may be the case, but it will require more precisely targeted investigations using new techniques, such as optogenetics, to confirm or deny this hypothesis.

Note added in proof: Since submission of this manuscript two computational models that incorporate grid cells and perform goal directed spatial navigation have, in fact, been published [86,87].

## Competing interests

The authors declare that they have no competing interests.

## Author contributions

CB and DB made equal academic contributions to this work and both participated in drafting the manuscript. Both authors read and approved the final manuscript.
